# Evaluation of medical and surgical decompression in patients with dysthyroid optic neuropathy

**DOI:** 10.1038/s41433-020-0897-x

**Published:** 2020-05-04

**Authors:** Aylin Garip Kuebler, Caroline Wiecha, Lukas Reznicek, Annemarie Klingenstein, Kathrin Halfter, Siegfried Priglinger, Christoph Hintschich

**Affiliations:** 1 Department of Ophthalmology, Ludwig-Maximilians-University, Munich, Germany; 2 Munich Cancer Registry, Institute for Medical Information Processing, Biometry, and Epidemiology, Ludwig-Maximilians-University, Munich, Germany

**Keywords:** Outcomes research, Thyroid diseases

## Abstract

**Purpose:**

To evaluate the effectiveness of steroid-pulse therapy and three-wall orbital decompression in patients with dysthyroid optic neuropathy (DON).

**Methods:**

Twenty-five patients (46 eyes) with a diagnosis of DON between 2008 and 2015 were included in the study. The first group (7 patients, 16 eyes) consisted of patients with a steroid-pulse treatment only and the second group (18 patients, 30 eyes) included patients with medical and surgical decompression.

**Results:**

Twenty patients were female; five patients were male. After the diagnosis of DON, all patients were treated with steroid-pulse treatment (intravenous 500 mg prednisolon twice/week for 4 weeks, 250 mg twice/week for 2 weeks) as a first-line treatment (medical decompression). In 30 eyes (18 patients) out of 46 eyes, (25 patients) an orbital decompression was needed to preserve the optic nerve function. In those therapy-resistant cases (surgical decompression group), the orbital decompression led to statistically significant improvements in best-corrected visual acuity (BCVA), protan and tritan value of the color vision (*p* = 0.007, *p* < 0.0001, *p* = 0.019, respectively, comparison of first visit to last visit).

**Conclusion:**

According to our data, the mild cases of DON with better initial visual acuity (in our case series mean: 0.3 logMAR) seem to respond well to steroid treatment. However, therapy-resistant cases with an impaired initial BCVA (in our case series, mean: 0.6 logMAR) seem to need the surgery to preserve the optic nerve function. In conclusion, this retrospective study confirms the effectiveness of surgical decompression in therapy-resistant cases of DON.

## Introduction

Graves’ orbitopathy (GO) is an immune-mediated inflammatory disease attributed to glycosaminoglycan depositions by fibroblasts into the retrobulbar tissues resulting in fibrosis [1]. The disease causes an enlargement of the extraocular muscles and increased volume of the orbital fat [2]. GO can be classified as mild, moderate to severe or sight threatening [3]. According to the Consensus Statement of the European Group on Graves’ Orbitopathy (EUGOGO), sight-threatening GO is described as dysthyroid optic neuropathy (DON) and/or corneal breakdown requiring immediate intervention to preserve vision [3]. The first line of treatment in DON involves steroid-pulse treatment, also known as medical decompression. Severe cases of DON, however, which do not respond to this initial therapy, are often treated with surgical orbital decompression.

Our goal was to evaluate the effectiveness of surgical decompression among patients that were resistant to the first line of therapy.

## Patients and methods

Herein we retrospectively studied the medical charts of 25 patients, who were treated in the Ludwig-Maximilians-University, Department of Ophthalmology, Section of Plastic and Reconstructive Surgery (Mathildenstrasse 8, 80336, Muenchen, Germany) between 2008 and 2015. All patients with a clinical diagnosis of DON were included in the study. We excluded only one patient, who was examined only once and thereafter refused to continue with further examinations. This retrospective, single-center study was approved by the institutional review board of Ludwig-Maximilians-University, Department of Ophthalmology. After obtaining approval, we reviewed the medical charts of a total of 25 patients. Of the 50 eyes of 25 patients, 46 eyes were diagnosed with DON and included in the study.

The diagnosis of DON was made if at least one of the following clinical findings and one of the additional diagnostic examinations were compatible with DON. Clinical findings comprised of (i) worsening of best-corrected visual acuity (BCVA) in comparison with the prior visit (≥2 lines), (ii) in cases where the first visit took place during a suspected diagnosis of DON, BCVA with pinhole being ≤0.6 (Snellen); but not owing to corneal problems or other pre-existing eye diseases; (iii) positive Swinging-Flash-Light-Test (SWFLT), and/or (iv) optic disc edema. Additional diagnostic examinations consisted of (i) visual field (VF) testing, (ii) color vision test by Arden, (iii) orbital computerized tomography (CT).

The first visit (Visit 1) was defined as the time of the clinical diagnosis of DON.

In every visit, ophthalmic examination included best-corrected visual acuity (BCVA) assessed with Snellen charts and documented as LogMAR function for statistical analysis, slit-lamp biomicroscopy, ocular motility, measurement of the eyelid position, measurement of proptosis with Hertel exophthalmometry, SWFLT and indirect ophthalmoscopy. Assessment of the activity of GO was done according to the EUGOGO recommendations with the clinical activity score (CAS) including 7 items at the first visit and 10 items at the following visits [4]. All patients had an orbital CT.

The characteristics of the patients are summarized in Table 1. All patients with DON were treated with intravenous (i.v.) pulses of 500 mg prednisolon (Solu Decortin: prednisolon 21-hydrogensuccinat) twice/week for 4 consecutive weeks, prednisolone was then reduced to 250 mg twice/week for the following 2 weeks (also known as medical decompression). The duration of the medical treatment was 6 weeks and the cumulative dose of prednisolone was 5 grams. In our case series, eight patients were also treated with an additional i.v. 1 gm prednisolone for 3 consecutive days owing to the severe clinical findings of DON in order to prevent complications of the disease.

**Table 1:** Baseline characteristics of the patients with Dysthyroid Optic Neuropathy (DON).

	Surgical decompression (*n* = 30 eyes) (*n* = 18 patients)	Medical decompression only (*n* = 16 eyes) (*n* = 7 patients)	*P-Value*
Female/Male	16/2	4/3	–
Age (mean)	55 (range 38–85)	62 (range 45–75)	0.862
Observation period [days] (mean)	303 (range 95–636)	361 (range 95–1601)	0.481
Prior treatment			
Radioiodine Treatment	5	3	–
Thyroidectomy prior to DON diagnosis	10	3	
Thyroidectomy after the diagnosis of DON	3	1	
None	1	1	
Systemic treatment of GO			
L-Thyroxin	11	2	–
Selenium (300 μgr/ daily)	4	0	
Carbimazole	2	0	
None	4	5	
Smoking status			
Current smoker	10	5	–
Ex-Smoker	3	0	
Never smoked	5	2	
Δ End of STX – Surgery [days]	75 (range −36–331)		–
CAS Grading (Visit 1) (median)	7/10 (range 3–8)	5/10 (range 4–8)	0.247
Intraocular Pressure (Visit 1) (mmHg)	17.00 (range 12–25)	17.50 (range 12–21)	0.268
fT3 (pmol/liter)	2.88 (range 0.90–3.90)	2.74 (range 2.26–3.30)	0.296
fT4 (pmol/liter)	1.56 (range 0.55–12.10)	1.19 (range 1.00–5.60)	0.370
TSH (mU/liter)	0.33 (range 0.01–7.90)	2.01 (range 0.01–15.20)	0.203
TSH-R (IU/liter)	17.40 (range 1.00–40.00)	6.60 (range 0.71–38.00)	0.333

*GO*: Graves’ Orbitopathy, *STX*: Steroid pulse treatment, *CAS*: Clinical Activity Score, *TSH*: Thyroid stimulating hormone, *TSH-R*: TSH receptor autoantibodies.

Premature intervention was called for when a surgical decompression with three-wall orbital decompression was needed during the steroid treatment owing to a worsening of the BCVA in Snellen chart accompanying other signs of optic neuropathy (such as worsening of VF, worsening of color vision, and/or insufficient improvement of BCVA during the medical decompression). In our case series, there were only three patients (four eyes) with worsening of the clinical symptoms during steroid treatment, which needed a surgical decompression as a premature intervention to prevent the loss of sight.

In this retrospective, observational study, patients were divided into two groups: The first group (medical decompression only) consisted of patients with a steroid-pulse treatment alone (7 patients, 16 eyes). The second group consisted of patients who received steroid treatment as a first-line treatment, but needed a surgical decompression after/during the steroid treatment due to worsening of symptoms/clinical findings (18 patients, 30 eyes). Owing to small sample size (three patients), we did not consider defining a third group (patients who underwent premature intervention with surgical decompression during the steroid-pulse treatment).

In all patients with DON or suspected DON, an automated perimetry with Humphrey Field Analyzer (Humphrey Instruments Inc., Dublin, CA) was performed. In statistical analysis, we only included the reliable VF examinations (where reliability was defined as <25% of fixation losses and <20% of false positive or negative responses). During VF testing, all patients had a best-corrected visual acuity with an adequate near correction.

In all patients, a computerized color vision test by Arden was performed. In this examination, a 21-inch color monitor was used to present the 10 random letters (A, E, H, M, O, T, U, V, X and Y) for 200 millisecond (ms) in the middle of the monitor to patients from a distance of 1.5 meters. The letters have an equal luminance with the background and can only be recognized owing to their difference in hue from the background. In our case series, protan and tritan were considered as pathological if the threshold was ≥8% [5]. The measurement of the intraocular pressure (IOP) was performed with Goldmann-Applanations-Tonometry.

The results and the development of the clinical examinations including VF, BCVA, SWFLT, Arden color test can be seen in Table 2.

**Table 2:** Development of the visual field, visual acuity, Swinging-flash-light-Test (SWFLT), Arden color test including protan and tritan values.

	Visit 1		Visit 2		Visit 3 (post)		Visit 4	
	Surgery	Steroid-Pulse only	Surgery	Steroid-Pulse only	Surgery	Steroid-Pulse only	Surgery	Steroid-Pulse only
Visual field								
Defects								
Yes	26	11	23	13	10	6	2	4
No	1	–	–	1	3	4	12	4
Missing	3	5	7	2	17	6	16	6
MD^a^	−7.2 (−14.9 to −3.5)	−10.2 (−15.9 to −5.3)	−8.6 (−14 to −4.3)	−7.0 (−11.3 to −4.6)	−5.7 (−20.8 to −3.8)	−4.1 (−11.0 to −2.5)	−2.3 (−3.2 to −0.4)	−3.1 (-5.8 to −0.5)
Visual acuity								
Visus^a^	0.26 (0.13–0.50)	0.57 (0.40–0.80)	0.20 (0.07–0.27)	0.63 (0.43–0.80)	0.32 (0.10–0.50)	0.63 (0.43–0.80)	0.50 (0.32–0.80)	0.90 (0.67–1.00)
Logmar^a^	0.6 (0.3–1.0)	0.3 (0.1–0.4)	0.7 (0.6 –11.5)	0.2 (0.1–0.4)	0.5 (0.3–1.0)	0.2 (0.1–0.4)	0.3 (0.1–0.5)	0.1 (0–0.2)
SWFLT								
Negative	23	14	21	14	27	16	28	16
Positive	7	2	9	2	3	–	2	–
Color test								
Abnormal	19	11	13	3	25	8	7	4
Normal	5	4	4	3	1	3	9	2
Missing	6	1	13	10	4	10	14	10
Protan^a^	37.00 (6.23–100.00)	9.90 (5.20–18.40)	38.56 (10.45–97.15)	8.00 (5.30–18.40)	15.40 (6.00–94.90)	5.80 (3.53–14.98)	6.70 (3.53–12.85)	8.40 (6.73–17.93)
Tritan^a^	45.7 (18.58–100)	26.30 (13.70–35.80)	60.85 (19.78–100)	26.90 (10.90–40.60)	62.80 (17.65–100)	15.65 (12.00–20.83)	13.70 (8.40–42.60)	17.35 (11.10–30.63)

Visit 1: Diagnosis of dysthyroid optic neuropathy (DON) and begin the treatment with steroid-pulse therapy.

Visit 2: The visit after the medical decompression (in three patients, this visit took place during the steroid-pulse treatment owing to worsening of symptoms).

Visit 3: for the medical decompression only group: the second follow-up visit after the steroid-pulse treatment. For the surgical decompression group: first follow-up visit after the surgery.

Visit 4: last visit of the patients.

^a^Median, 25–75th percentile shown.

## Statistical analysis

The baseline cohort demographics is described by median±range or the 25th and 75th percentiles (abbreviated as the interquartile range; IQR) in Table 1.

The subsequent group comparisons (medical decompression only and surgical decompression) in BCVA, VF, Arden color test (protan and tritan) were all conducted using the Mann–Whitney *U* test. The longitudinal analysis of the observed parameters (BCVA, MD of the VF, tritan, protan) was conducted independently for each treatment. Specifically, the changes in measured values from one visit to the next, in addition to the change from visit 1 to the last visit, were evaluated separately for the two groups (medical decompression only and surgical decompression). The Friedman test was used for the overall assessment of the repeated measurements and the Wilcoxon-signed-rank test with Bonferroni correction was employed for individual time-point comparisons within each treatment group. For all two-sided *p* values, a significance level of 0.05 was used. All data were recorded and analyzed using SPSS V.25.0 (Spss Inc, Chicago, IL, USA).

## Results

Of the 25 patients (46 eyes) included in the study, 21 patients had a bilateral involvement. We divided the patients into two groups and conducted analyses separately for each group. In the initial descriptive analyses, however, we compared the two groups with one another in order to establish the different characteristics of patients that ended up in each group.

The first group of patients (medical decompression: steroid treatment only) included 16 eyes, which were only treated with steroid-pulse treatment. The second group of patients (medical and surgical decompression) included 30 eyes, which were treated initially with steroid-pulse treatments and then with three-wall-orbita decompression. Eight patients in this group (16 eyes) were also treated with i.v. Solu Decortin 1000 mg for 3 days in order to prevent potential blindness prior to the steroid-pulse treatment. Interestingly, all of these patients ended up having surgical decompression despite receiving high doses of pulse treatment and steroid.

After the first visit, all patients were treated with eight pulses prednisolone as described. The median BCVA (LogMAR) at the first visit was 0.6 with the IQR lying between 0.3 and 1.0 in the surgery group and 0.3 (IQR 0.1–0.4) in the group of patients with medical decompression only. Thirty-nine eyes (84.8%) had an impaired BCVA (≤0.6(LogMAR). The surgery group had a statistically significant worse initial BCVA at visit 1 compared to the medical decompression group (*p* = 0.003).

At the time of the diagnosis of DON (visit 1), 37 out of 38 eyes showed an altered automated VF (97%). The median of the mean deviation (MD) was −10.2 (IQR between −15.9 and −5.3) in the group with medical decompression only and −7.2 (IQR between −14.9 and −3.5) in the group with surgical decompression. There was no statistically significant difference between the groups in MD of the VF at visit 1 (*p* = 0.710).

At visit 1, a total of 9 out of 46 eyes had a pathological SWFLT, and 30 of 39 eyes had an abnormal Arden color test (protan/tritan threshold being ≥8%). The median protan value was 37% (IQR 6.23–100%) in the group with surgical decompression and 9.90% (IQR 5.20–18.40%) in the group with medical decompression only. The median tritan value was 45.7% (IQR 18.58–100) in the group with surgical decompression and 26.30% (IQR 13.70–35.80%) in the group with medical decompression only. The difference in protan between the two groups was statistically significant (*p* = 0.040), whereas the difference in measured tritan values was not (*p* = 0.075). The median CAS was 7 (IQR 5– 8) in the group with surgical decompression and 5 (IQR 5–7) in the group with medical decompression only; the difference in initial CAS between the two groups at visit 1 was not statistically significant (*p* = 0.247). The mean IOP during visit 1, measured with Goldmann tonometry, was 17.00 mmHg (IQR 15.00–18.00) in patients with surgical decompression and 17.50 mmHg (IQR 16.00–19.25) in patients with medical decompression only (*p* = 0.268). Only 2 of 46 eyes (4.4%) had an optic disc swelling at the first presentation (visit 1).

The second visit was described as the visit after the medical decompression, however, as mentioned before, three patients (four eyes) needed a surgical decompression before finishing the steroid treatment. The mean time interval between the end of the steroid treatment and visit 2 was 75 days.

At visit 2, the mean BCVA with LogMAR was 0.7 (IQR 0.6–11.5) in patients with surgical decompression and 0.2 (IQR 0.1–0.4) in patients with medical decompression only. At visit 2, there were 23 eyes with an altered VF in the surgical decompression group as opposed to 13 eyes in the group with medical decompression only. The median MD was −8.6 (IQR between −14 and −4.3) in the surgery group and −7.0 (IQR between −11.3 and −4.6) in the medically treated group. At visit 2, there were 16 eyes (13 in the surgical decompression group and 3 in the medically treated group) with a pathological Arden color test. The median protan value was 38.56% (IQR 10.45–97.15) in the group with surgical decompression and 8.00% (IQR 5.30–18.40) among the patients with medical decompression only.

After visit 2, in cases with insufficient recovery or worsening of BCVA, visual field, and/or color vision, surgical decompression was performed in order to preserve the optic nerve function. The surgical procedure was performed by a single surgeon (CH). Thirty-eyes underwent a three-wall orbital decompression.

Visit 3 was defined for the group with surgical decompression as the visit after the surgery, and for the patients with medical decompression only, as the second visit following the steroid-pulse treatment.

Out of 46 eyes, 30 eyes underwent a surgical orbital decompression. The time interval between starting of the steroid treatment and decision to operate could be calculated in 28 eyes and had a median of 98.6 days (range: 36–331 days). The median time interval between surgery and visit 3 was 26.5 days (range: 3–215 days) in the group with surgical decompression. The median time interval between the end of the steroid-pulse treatment and the visit 3 was 126 days (range: 60–301 days) in the group with medical decompression only.

In the comparison of the last visit (visit 4) to the first visit (visit 1), there was a significant improvement of the BCVA (LogMAR) in both patients receiving surgical decompression and those with medical decompression only (*p* = 0.007 and *p* = 0.045, respectively). In the surgical decompression group, there was a statistically significant improvement of protan and tritan values of the color vision (*p* < 0.0001 and *p* = 0.019, respectively). Yet, the improvement of the MD of the VF was not statistically significant for this group (*p* = 0.252).

In the medical decompression only group, there was a statistically significant improvement in terms of MD of the VF (*p* = 0.022). Interestingly, the group showed no statistically significant improvement in terms of color vision, as measured by protan and tritan (*p* = 0.194 and *p* = 0.97, respectively).

Comparing one group with the other (surgical decompression vs medical decompression) in the development of the BCVA, MD of the VF, protan and tritan value of the color vision revealed interesting findings. At visit 1, in terms of BCVA and protan value, the surgical decompression group had a statistically significant worse initial condition compared with group that received medical decompression only (*p* = 0.003 and *p* = 0.04, respectively). However, in terms of MD of the VF, tritan value, and CAS, there were no statistically differences between the two groups at the first visit (*p* = 0.710, *p* = 0.075 and *p* = 0.247, respectively).

Furthermore, comparing the last visit (visit 4) to the first visit (visit 1), there was a significantly higher improvement of the BCVA (LogMAR) in patients with surgical decompression relative to those with medical decompression alone (*p* = 0.045). The same trend was also seen in VF examination. There was a significantly higher improvement of MD of the visual field examination (*p* = 0.022), protan (*p* = 0.02) and tritan (*p* = 0.019) in the surgical compression group compared with the medical decompression only patients. The comparisons between the two groups in BCVA and protan values should be interpreted with caution, because the surgery group had statistically significant worse initial situation in terms of BCVA and protan relative to the medical decompression group. Yet, the findings attest to the success of surgical decompression in cases where medical decompression is not sufficient to restore patients’ vision.

The changes in BCVA (Supplementary Fig. 1a), MD of the VF examination (Supplementary Fig. 1b), protan (Supplementary Fig. 1c) and tritan value of the color vision (Supplementary Fig. 1d) can be seen in the supplementary information.

## Discussion

In DON, treatment aims to reduce the pressure on the optic nerve by lessening the volume of the orbital content, and thereby decreasing the ongoing inflammation. At present, there is no standardized, evidence-based treatment algorithm for DON. The oldest therapy involves Glucocorticoids (GCs), which can be applied locally (retrobulbar or subconjunctival), orally or i.v. [6]. In addition, there are many schedules using steroid application. Guy et al. [7] reported on the successful treatment of five DON patients with 1 g daily of i.v. methylprednisolone (MP) for 3 consecutive days, suggesting the benefits of pulse therapy in the initial management of the condition.

Mourits et al. [8] later claimed that delaying surgical orbital decompression, and trying the MP treatment first, does not affect the final visual outcome. Still, 61% of the patients had to undergo orbital decompression because of persistent or recurrent DON within one week to six months after i.v. MP.

Kahaly et al. [9] subsequently reported on a randomized, single-blinded study of 70 patients with active and severe GO, showing improved results with i.v. MP (0.5 g/week iv MP, then 0.25 g/week iv MP −6 weeks each-, cumulative dose: 4.5 g) compared with a treatment with oral prednisolone (starting doses: 100 mg/day, tapered by 10 mg/week for 12 weeks, cumulative dose: 4 g). The authors also established the benefit of i.v. treatment in patients with constant diplopia in primary gaze, where the number of cases with optic neuropathy markedly declined after i.v. treatment.

In light of recent studies, we can conclude that, owing to the rare side effects and statistically better results, the i.v. GC is more beneficial than oral prednisolone [10]. This finding is important, but still not sufficient for us to address DON completely. Despite the existence of many prospective and retrospective studies on treatment of DON, we still do not know the appropriate frequency, duration and dosage of i.v. MP. Our study contributes to this growing line of empirical work establishing the importance of i.v. MP in the treatment in DON.

Another critical question in the literature is whether surgical orbital decompression can be an effective alternative to i.v. MP application (and thus used as a first-line treatment) in patients with DON. To answer this question, Wakelkamp et al. [11] conducted a randomized, controlled study including 15 patients with DON. Despite relying on a small group of patients, the study established that an orbital decompression surgery is not recommended as a first-line treatment in DON.

Another recent study by Curro et al. [12] included 24 patients (40 eyes) with DON, who were treated with a high dose of i.v. MP (0.5 or 1 g pulsed i.v.–MP daily for 3 consecutive days, repeated after 1 week, and then tapered off either orally or intravenously). The authors speculated that the i.v. MP is effective, however, in almost half of the cases, surgery was needed owing to an inadequate response to steroids or relapse of DON. Our results confirm these findings with a larger sample size, and explain why some surgeons still favor orbital decompression in cases that do not respond to the medical treatment.

Garrity et al.’s study is instructive on orbital decompression. The authors presented a large case series of 428 patients with severe GO (217 patients, 50.7% with DON), who were treated with transantral orbital decompression [13]. The results showed improvement or stabilization of the visual acuity in 402 of 453 eyes (89%). The authors concluded that transantral orbital decompression effectively reduces proptosis and usually corrects optic neuropathy. Our results are in line with Garrity et al.’s findings. Although our sample size is smaller, our study has the additional advantage of including all patients of DON, not just those receiving surgical treatment.

Our study set out to combine to lines of research in existing work. One line considers the effectiveness of medical treatment of DON with steroids (Guy et al. 1989, Kahaly et al. 2005), whereas the other evaluates surgery as a first-line treatment (Wakelkamp et al. 2005). Few studies consider both treatments in tandem (e.g., Mourits et al. 2001; Curro et al. 2014). We add to this growing line of research, and evaluate both medical and surgical treatment of DON.

Our results showed that both treatments (medical decompression and surgical decompression) yielded improvements in BCVA in their respective patient groups. Specifically, for 30 out of 46 eyes, medical decompression did not lead to sufficient recovery, and surgical decompression was used. Eight patients in this group were treated with high doses of i.v. steroids (1000 mg prednisolone for 3 days) prior to the 6-week scheme (500 mg twice/week for 4 weeks and 250 mg twice/week for 2 weeks) owing to the potential visual loss. These patients all needed surgery despite having received the maximum medical treatment (steroid-pulse treatment and the 1000 mg of prednisolone for 3 days additionally).

These findings highlight two points. First, mild cases of DON with a better initial visual acuity (in our case series, median: 0.3 ogMAR) seem to respond well to steroid treatment, sparing patients surgery. Second, therapy-resistant cases with a worse initial BCVA (in our case series, median: 0.6 logMAR) still need surgery to preserve the optic nerve function. But, thankfully, those cases respond well to the surgery, and despite having a worse initial condition of BCVA, end up with a statistically significant higher value of BCVA post-operation (*p* < 0.0001 visit 2 vs visit 4), which they did not show immediately after the medical treatment.

One limitation of our study is small sample size that is attributable to the rarity of the disease. Although our sample of 25 patients is small looking at studies evaluating only a single treatment method (for example, Garrity et al. 1993), it is comparable to studies considering both, the medical and surgical treatment of those patients (such as Mourits et al. 2001). Another limitation, common to prior work, is the retrospective study design, which might lead to missing data.

For future work undertaking similar comparisons, it is important to highlight the uneven initial conditions for the two groups (medical decompression and surgical decompression) in our analysis. The surgery group consisted of the therapy-refractory cases with a significantly worse condition of BCVA and protan value of the color vision. Although the two groups varied on BCVA and protan, they showed no statistically significant differences in MD of the VF and tritan values. Future work could also evaluate whether such differences and similarities could better guide the sorting of patients into different first-line treatment regimes.

In conclusion, this retrospective study confirms the effectiveness of medical decompression in mild cases of DON as well as the effectiveness of surgical decompression in therapy-refractory cases. For a better understanding of the effectiveness of medical and surgical treatment of DON, more prospective randomized studies are needed.

### Summary

#### What was known before

- The first line of treatment in DON involves steroid-pulse treatment, also known as medical decompression.
- Up to date, there has been many publications, speculating medical treatment could spare the surgery.
- Currently, there is no standardized, evidence-based treatment algorithm for DON.
- Our goal was to evaluate the effectiveness of surgical decompression among patients that were resistant to the first line of therapy.

#### What this study adds

- Our findings highlight two points: first, mild cases of DON with a better initial visual acuity (in our case series, median: 0.3 logMAR) seem to respond well to steroid treatment, sparing patients surgery.
- Second, therapy-resistant cases with a worse initial BCVA (in our case series, median: 0.6 logMAR) still need surgery to preserve the optic nerve function.
- But, thankfully, those cases respond well to the surgery, and despite having a worse initial condition of BCVA, end up with a statistically significant higher value of BCVA post-operation (*p* < 0.0001 visit 2 vs visit 4), which they did not show immediately after the medical treatment.
- In our case series, 30 eyes (18 patients) out of 46 eyes, (25 patients) an orbital decompression was needed to preserve the optic nerve function.
- According to our data, in therapy-refractory cases of DON, surgical decompression remains inevitable.

## Supplementary information

Supplementary material
