# Correction: Evaluation of medical and surgical decompression in patients with dysthyroid optic neuropathy

**DOI:** 10.1038/s41433-021-01683-y

**Published:** 2021-08-19

**Authors:** Aylin Garip Kuebler, Caroline Wiecha, Lukas Reznicek, Annemarie Klingenstein, Kathrin Halfter, Siegfried Priglinger, Christoph Hintschich

**Affiliations:** 1 Department of Ophthalmology, Ludwig-Maximilians-University, Munich, Germany; 2 Munich Cancer Registry, Institute for Medical Information Processing, Biometry, and Epidemiology, Ludwig-Maximilians-University, Munich, Germany

**Keywords:** Outcomes research, Thyroid diseases

Correction to: *Eye*

10.1038/s41433-020-0897-x

The article “Evaluation of medical and surgical decompression in patients with dysthyroid optic neuropathy”, written by Aylin Garip Kuebler et al., was originally published Online First without Open Access. After publication in volume 34, issue 9, page 1702–1709 the author decided to opt for Open Choice and to make the article an Open Access publication. Therefore, the copyright of the article has been changed to © Author(s) 2021 and the article is forthwith distributed under a Creative Commons Attribution 4.0 International License, which permits use, sharing, adaptation, distribution and reproduction in any medium or format, as long as you give appropriate credit to the original author(s) and the source, provide a link to the Creative Commons licence, and indicate if changes were made. The images or other third party material in this article are included in the article’s Creative Commons licence, unless indicated otherwise in a credit line to the material. If material is not included in the article’s Creative Commons licence and your intended use is not permitted by statutory regulation or exceeds the permitted use, you will need to obtain permission directly from the copyright holder. To view a copy of this licence, visit http://creativecommons.org/licenses/by/4.0. Open access funding enabled and organized by Projekt DEAL.

Funder Name: Ludwig-Maximilians-Universität München (1024).

