# Supplementary material for: Evaluation of medical and surgical decompression in patients with dysthyroid optic neuropathy
Source: Eye (Lond). 2020 May 4;34(9):1702–9. doi: 10.1038/s41433-020-0897-x (PMC7608314; doi:10.1038/s41433-020-0897-x)
Supplement: Supplementary file 1 — Supplementary material [file 41433_2020_897_MOESM1_ESM.docx]

**Supplementary Figures:**

**Figure 1a**. The changes in Best Corrected Visual Acuity (BCVA) (LogMAR)

**Figure 1b.** The changes of Mean Deviation (MD) in visual field examination

**Figure 1c.** The changes of protan value in colour vision by Arden

**Figure 1d.** The changes in tritan value in colour vision by Arden
